# The Prevalence and Risk Factors Associated with Iodine Deficiency in Canadian Adults

**DOI:** 10.3390/nu14132570

**Published:** 2022-06-21

**Authors:** Stellena Mathiaparanam, Adriana Nori de Macedo, Andrew Mente, Paul Poirier, Scott A. Lear, Andreas Wielgosz, Koon K. Teo, Salim Yusuf, Philip Britz-Mckibbin

**Affiliations:** 1 Department of Chemistry and Chemical Biology, McMaster University, Hamilton, ON L8S 4L8, Canada; mathis2@mcmaster.ca (S.M.); adrianandm@gmail.com (A.N.d.M.); 2 Departamento de Química, Universidade Federal de Minas Gerais, Belo Horizonte 31270-901, MG, Brazil; 3 Population Health Research Institute, McMaster University and Hamilton Health Sciences, Hamilton, ON L8L 2X2, Canada; Andrew.Mente@phri.ca (A.M.); Koon.Teo@phri.ca (K.K.T.); Salim.Yusuf@phri.ca (S.Y.); 4 Faculté de Pharmacie, Institut Universitaire de Cardiologie et de Pneumologie de Québec, Université Laval, Québec City, QC G1V 4G5, Canada; Paul.Poirier@criucpq.ulaval.ca; 5 Faculty of Health Sciences, Simon Fraser University, Burnaby and Division of Cardiology, Providence Health Care, Vancouver, BC V5A 1S6, Canada; SLear@providencehealth.bc.ca; 6 University of Ottawa Heart Institute, Ottawa, ON K1Y 4W7, Canada; andreas.wielgosz@uottawa.ca

**Keywords:** iodine deficiency, iodine deficiency disorders, urinary iodine concentration, urinary iodine excretion, nutrition, dietary intake, epidemiological studies, iodine uptake inhibitors

## Abstract

Iodine is a trace micronutrient that is critical for normal thyroid function and human health. Inadequate dietary intake is associated with cognitive impairment, infertility, growth retardation and iodine deficiency disorders in affected populations. Herein, we examined the prevalence of iodine deficiency in adults (median age of 61 years) based on the analysis of 24 h urine samples collected from 800 participants in four clinical sites across Canada in the Prospective Urban and Rural Epidemiological (PURE) study. Urinary iodide together with thiocyanate and nitrate were measured using a validated capillary electrophoresis assay. Protective/risk factors associated with iodine deficiency were identified using a binary logistic regression model, whereas daily urinary iodine concentration (24 h UIC, μg/L) and urinary iodine excretion (24 h UIE, μg/day) were compared using complementary statistical methods with covariate adjustments. Overall, our Canadian adult cohort had adequate iodine status as reflected by a median UIC of 111 μg/L with 11.9% of the population <50 μg/L categorized as having moderate to severe iodine deficiency. Iodine adequacy was also evident with a median 24 h UIE of 226 μg/day as a more robust metric of iodine status with an estimated average requirement (EAR) of 7.1% (< 95 μg/day) and a tolerable upper level (UL) of 1.8% (≥1100 μg/day) based on Canadian dietary reference intake values. Participants taking iodine supplements (OR = 0.18; *p* = 6.35 × 10^−5^), had greater 24 h urine volume (OR = 0.69; *p* = 4.07 × 10^−4^), excreted higher daily urinary sodium (OR = 0.71; *p* = 3.03 × 10^−5^), and/or were prescribed thyroxine (OR = 0.33; *p* = 1.20 × 10^−2^) had lower risk for iodine deficiency. Self-reported intake of dairy products was most strongly associated with iodine status (*r* = 0.24; *p* = 2.38 × 10^−9^) after excluding for iodine supplementation and T4 use. Participants residing in Quebec City (OR = 2.58; *p* = 1.74 × 10^−4^) and Vancouver (OR = 2.54; *p* = 3.57 × 10^−4^) were more susceptible to iodine deficiency than Hamilton or Ottawa. Also, greater exposure to abundant iodine uptake inhibitors from tobacco smoking and intake of specific goitrogenic foods corresponded to elevated urinary thiocyanate and nitrate, which were found for residents from Quebec City as compared to other clinical sites. Recent public health policies that advocate for salt restriction and lower dairy intake may inadvertently reduce iodine nutrition of Canadians, and further exacerbate regional variations in iodine deficiency risk.

## 1. Introduction

Iodine is an essential trace micronutrient in human health used in the biosynthesis of thyroid hormones, which regulate cellular metabolism, growth, and development throughout the lifespan [1,2]. Iodine deficiency remains a global public health concern since it increases neonatal mortality and is a preventable cause of cognitive impairment and developmental delays in children [3,4]. Although reproductive age women and school-aged children represent vulnerable groups [5], thyroid disorders attributed to mild to moderate iodine deficiency are also implicated in chronic disease burden in adults and older persons [6,7], which include immunomodulatory effects on immune function [8]. Nonetheless, remarkable progress has been made in expanding universal salt iodization programs to reduce the prevalence of iodine deficiency disorders worldwide [9,10]. However, several developing and developed countries still suffer from persistent iodine deficiency (e.g., Cambodia, Russia, Israel) or excessive iodine intake (e.g., South Korea, Cameroon, Columbia) that may also contribute to deleterious health outcomes [11,12]. Changing dietary patterns in contemporary societies have also altered the efficacy of iodine prophylaxis [13] through restriction of sodium intake to reduce blood pressure [14] along with increased consumption of processed foods using non-iodized salt [15]. As a result, continuous surveillance is critical to ensure optimal iodine status and to evaluate the impact of recent public health interventions, such as iodine fortification of staple foods (e.g., bread) adopted in Australia [16].

Food frequency questionnaires for estimating iodine intake in populations are limited in capturing the variable content of iodine in similar foods, which may also not be reported in national food composition databases [17]. For instance, cow’s milk represents a major source of dietary iodine that varies widely in retail products based on feed composition, iodine supplementation and teat-dipping sanitation practices using iodophors [18]. As most iodine consumed is excreted as iodide, the median urinary iodine concentration (UIC) offers an objective indicator of iodine intake while also serving as a surrogate measure of the prevalence of goiter and thyroid nodules in a population [19]. However, up to ten repeat urine spot samples or 24 h urine collection is needed to reliably estimate individual iodine status [20]. According to the World Health Organization (WHO), adequate iodine intake for adults is indicated by a median UIC within 100 to 199 μg/L along with a low rate (<20%) of moderate to severe iodine deficiency (<50 μg/L) [9,21]. Higher thresholds for iodine deficiency are designated for children and pregnant/lactating women [22]. Daily iodine intake (μg/day) can be estimated from 24 h urinary iodine excretion (UIE) from spot urine iodine concentrations after correction for age, sex, ethnicity and/or anthropometric dependent creatinine adjustments. These adjustments correct for between-subject variations in urine fluid volume and muscle mass [23,24,25]. As urinary concentrations of iodide reflect recent dietary intake of iodine-containing foods or beverages, other factors can also affect iodine status determination when using spot urine samples, such as skipping breakfast prior to morning urine sampling [26]. Although less convenient to collect, 24 h urine samples offer greater reproducibility and accuracy than spot urine samples and are the preferred method for assessing iodine status in epidemiological studies [27]. 

Although populations in North America are considered to have adequate iodine status [11], differences remain across certain demographic groups and regions [28]. For instance, a median UIC of 134 μg/L from spot urine samples was reported in Canadian households surveyed from 2009 to 2011, with about 22% and 7% of Canadians at risk for mild and moderate iodine deficiency, respectively [29]. Furthermore, iodine intake is frequently inadequate for women of child-bearing age with recommendations for use of a daily multivitamin-mineral supplement containing iodine during pregnancy and breastfeeding [30]. The risk for iodine deficiency is also dependent on environmental exposure to perchlorate, thiocyanate and nitrate that competitively inhibit active iodine uptake via the sodium-iodine symporter expressed in the intestine and thyroid gland [31]. To date, few epidemiological studies have examined the iodine status and environmental exposure to iodine uptake inhibitors in diverse populations across different regions [32]. In this work, we examined the iodine status in 24 h urine specimens collected from participants (*n* = 800) in the Prospective Urban and Rural Epidemiological (PURE) study [33]. A validated method based on capillary electrophoresis (CE) was used for simultaneous analysis of urinary iodide, nitrate and thiocyanate after a simple dilution step [34,35]. Our study aimed to identify risk/protective factors associated with iodine deficiency for participants residing in four communities across Canada reflecting differences in dietary habits, smoking status, and other environmental exposures.

## 2. Experimental

### 2.1. Study Design, Participant Eligibility, and 24 h Urine Sampling 

Our cross-sectional study included a subset of participants from the PURE-24USE (PURE-24 Hour Urinary Sodium Excretion Survey) study [34] who were recruited from January 2012 to December 2013. This cohort included adults aged from 36 to 83 years (median of 61 years) living in four sites across Canada, Hamilton (*n* = 217), Vancouver (*n* = 200), Quebec City (*n* = 200), and Ottawa (*n* = 183). Ethical approval was provided by local research ethics boards at the four clinical sites, and all participants from each site provided signed informed consent [33]. Participants were excluded if they were diagnosed with a debilitating disease, required food restrictions due to chronic illness, as well as pregnant or breastfeeding women who are contraindicated from ingestion of *para*-aminobenzoic acid (PABA). A standardized procedure, necessary supplies, and detailed instructions for collecting 24 h urine specimens were provided to all PURE participants as described elsewhere [33]. Participants aged under 65 years also ingested a PABA tablet (80 mg) at each of the three meals to verify adherence to 24 h urine sampling [36]. Urine samples were considered authentic for subsequent analysis when recovery was >85% for ingested PABA, and urinary 24 h creatinine excretion was within reference intervals for men (995 to 2489 mg) and women (509 to 1810 mg) [34]. However, older participants (>65 years) were exempted from PABA screening due to their delayed renal clearance [36]. On completion of collection, study staff measured and recorded the 24 h urine volume, thoroughly mixed the collection, and retained 2 mL aliquots that were stored frozen at −70°C within the Clinical Trials and Clinical Research Laboratory in Hamilton General Hospital [37]. 

### 2.2. PURE Participants and Self-Reported Dietary Intake

At each participant’s study visit, a standardized questionnaire was used to assess anthropogenic parameters, personal medical history, smoking status, alcohol intake, physical activity, and use of prescription medications (e.g., Levothyroxine or Synthroid, T4) and vitamin-mineral supplements [33]. Participants also completed a short questionnaire of salt exposures from foods consumed over the 24 h period when their urine sample was collected. Self-reported food intake was recorded by participants according to the type of product consumed with emphasis on assessment of sodium and/or potassium content in the PURE-24USE study [33]. In this work, foods associated with iodine nutrition and goitrogen intake were evaluated [38,39] including salty foods, dairy, eggs, fish, breads and cereals, meats (e.g., red, white, processed), processed foods, fruits, and various vegetables (e.g., green leafy, cruciferous, dark yellow). The amount of specific food products consumed daily were estimated as g/day unless otherwise indicated.

### 2.3. Iodide, Thiocyanate, Nitrate and Sodium Determination in 24 h Urine Samples 

Urine samples were analyzed for iodide that is the predominant species of iodine excreted in urine, as well as nitrate and thiocyanate using a recently validated assay based on capillary electrophoresis with UV absorbance detection [34,35]. Urinary sodium concentrations were measured by indirect potentiometry using a Beckman Coulter UniCel DxC600 Synchron Clinical System [33]. All chemical reagents were purchased from Sigma-Aldrich Inc. (Oakville, ON, Canada) unless otherwise stated. Briefly, frozen aliquots of urine were thawed slowly to room temperature and then diluted two-fold in deionized water containing an internal standard, 1,5-naphthalene disulfonic acid (NDS, 40 μM). In some cases, hydrated urine samples with low ionic strength were diluted two-fold in a simulated urine matrix solution comprised of 100 mM sodium chloride and 10 mM sodium sulfate for matrix matching purposes. All diluted urine samples were vortexed and centrifuged prior to analysis. Quality control (QC) samples based on a pooled urine sample from all PURE-24USE participants were used for assessment of technical precision. All CE separations were performed using a P/ACE™ MDQ system with UV absorbance detection (SCIEX, Framingham, MA, USA). Unmodified fused-silica capillaries were purchased from Polymicro Technologies Inc. (Phoenix, AZ, USA) with an internal diameter of 75 μm and total and effective (to detector window) capillary length of 60 cm and 50 cm, respectively. The background electrolyte (BGE) was composed of 180 mM lithium hydroxide, 180 mM phosphoric acid, 46 mM α-cyclodextrin with a pH of 3.0 [35]. New capillaries were initially conditioned by flushing with methanol, deionized water, 1.0 M lithium hydroxide (5 min each), and then background electrolyte (BGE) for 20 min using a rinse pressure of 20 psi (138 kPa). The CE separations were performed at 25 °C under reversed polarity with an applied voltage of −18 kV, and UV absorbance was monitored at 226 nm (for iodide, nitrate and thiocyanate) and 288 nm (for NDS). Prior to each analysis, the capillary was flushed with the BGE for 3 min at 20 psi (138 kPa) followed by a long sample injection via hydrodynamic pressure for 80 s at 0.5 psi (3.4 kPa). At the start of each day, a blank sample, a calibrant mixture, and a QC were analyzed by CE prior to a randomized analysis of a batch of individual urine samples with a QC sample repeatedly analyzed after every batch of ten runs. At the end of each day, the capillary was flushed with deionized water for 5 min and the inlet and outlet ends of the capillary were stored in vials containing deionized water overnight. Calibration curves were performed for iodide, nitrate and thiocyanate by CE, where their integrated peak areas were normalized to NDS as the internal standard. Urinary iodide, nitrate and thiocyanate concentrations from PURE-24USE participants were reported in terms of their absolute concentrations (μg/L or mg/L) or daily excretion amounts based on total volume of 24 h urine collected (μg/day or mg/day) that forgoes the need for creatinine adjustment. Dietary iodine intake estimates for PURE were derived from measured daily excretion amounts and adjusted by an iodine bioavailability of 92% [39]. Missing data following analysis of all 24 h urine samples were 0%, 2%, and 11% for nitrate, iodide and thiocyanate, respectively, if below method detection limits (*S*/*N* = 3; 0.020 μmol/L or 2.5 μg/L for iodide) or as a result of matrix interferences. For iodide non-detects, a missing value replacement was used corresponding to the lowest concentration measured in the cohort divided by 3.

### 2.4. Statistical Analysis

Descriptive statistics, box-whisker plots, and pair-wise Spearman rank correlations for urinary iodide, nitrate, thiocyanate and sodium concentrations and their equivalent daily excretion amounts were performed using MedCalc 12.5 statistical software (MedCalc Software Ltd, Ostend, Belgium). Routine data processing for population stratification (quintiles) and least-squares linear regression for calibration curves of urinary anions measured by CE were performed using Microsoft Excel (Redmond, WA, USA). Representative electropherograms and control charts were plotted using Igor Pro 5.04B (Wavemetrics Inc., Lake Oswego, OR, USA). Protective and risk factors associated with iodine deficiency (<100 µg/L or <150 µg/day) were evaluated using a binary multivariate logistic regression with and without adjustment for covariates. An odds ratio (OR) < 1.0 corresponds to a positive association with the outcome (i.e., protective factor against iodine deficiency), whereas an OR > 1.0 indicates a negative association to the outcome (i.e., risk for iodine deficiency). Also, analysis of covariance (ANCOVA) was performed on *log*-transformed data using SPSS 23.0 statistical software (IBM SPSS, Chicago, IL, USA). Statistical tests comparing iodine status among PURE participants as a function of regional site, iodine supplementation, T4 prescription, and dietary intake of specific foods were performed unadjusted and adjusted for covariates, including age, sex, body mass index (BMI), total caloric intake, current smoking, alcohol use, education, and diet quality (Alternative Healthy Eating Index, AHEI score) unless otherwise noted. Statistical significance was set at *p* < 0.05 with a Bonferroni correction used for multiple comparisons.

## 3. Results

### 3.1. PURE Cohort Characteristics and CE Method Performance 

Table S1 summarizes the cohort characteristics of this cross-sectional study comprising 800 PURE-24USE participants from four regional sites across Canada who completed 24 h urine sampling and a short questionnaire during their clinical visit. Overall, a sex-balanced cohort (females, *n* = 412; males, *n* = 388) of overweight Canadian adults with a mean age of (60 ± 9; 36 to 83) years and BMI of (28.1 ± 5.7; 16.3 to 59.4) kg/m^2^ were recruited primarily from urban (~ 86%) regions of Vancouver, Hamilton, Ottawa and Quebec City. Also, about 25% of PURE-24USE participants had high blood pressure based on a resting systolic blood pressure >140 mm Hg and/or diastolic blood pressure >90 mm Hg [33]. We searched the composition of all vitamin/mineral supplements reported to be taken by PURE participants and verified that iodine-containing supplements were consumed by 12.9% (*n* = 103). Additionally, 7.6% were prescribed thyroxine (T4, *n* = 61), and 7.4% (*n* = 59) were current smokers. However, participants with debilitating chronic diseases, restrictive diet requirements and pregnant women were excluded. All urine samples from participants were diluted minimally and then directly analyzed by CE with UV absorbance detection, which allowed for determination of iodide, as well as nitrate and thiocyanate within 10 min (Figure S1). Good technical precision was achieved following intermittent analysis of a pooled QC urine sample (*n* = 93) with a mean coefficient of variance (CV) < 8% as depicted in control charts. Table S2 lists the figures of merit of the CE method used for reliable quantification of iodide, nitrate and thiocyanate in 24 h urine samples from the PURE-24USE study.

### 3.2. Iodine Nutritional Status of Canadian Adults from PURE-24USE

**Figure 1:**
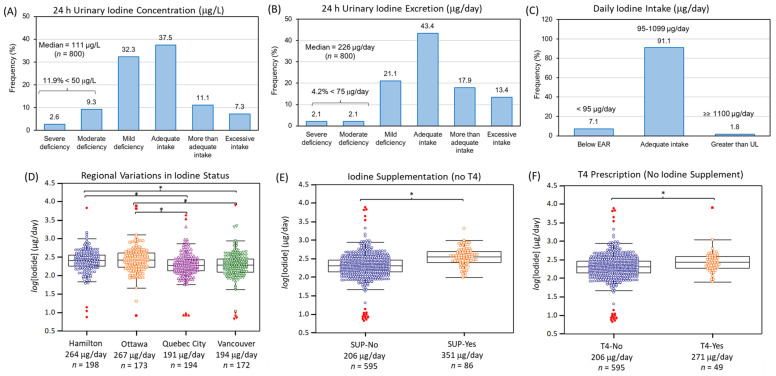
The iodine status of participants from the PURE-24USE study (*n* = 800) based on (**A**) 24 h UIC (μg/L) and (**B**) 24 h UIE (μg/day). Median iodine concentrations indicated iodine adequacy (111 μg/L or 226 μg/day, where error is ±IQR) with a low prevalence of moderate to severe iodine deficiency in the population (<20%). (**C**) Daily iodine intake confirmed only a small fraction of adult Canadians in our cohort were below EAR (7.1%, <95 μg/day) or greater than UL (1.8%, ≥1100 μg/day). (**D**) Regional variations in iodine nutrition were found with residents from Quebec City and Vancouver having lower iodine status than Hamilton or Ottawa (*F* = 8.80, *p* = 9.82 × 10^−6^, *n* = 737; where * *p* < 0.014 for pairwise comparisons). Participants who (**E**) reported use of multivitamin supplements containing iodine, but no T4 (*F* = 42.3, *p* = 1.52 × 10^−10^, *n* = 681), or (**F**) were prescribed T4, but not taking iodine supplements (*F* = 9.71, *p* = 1.91 × 10^−3^, *n* = 644) had greater iodine status than controls. All ANCOVA were adjusted for age, sex, BMI, total caloric intake, AHEI score, education, alcohol use, and smoking status.

Data distribution for urinary iodide, nitrate and thiocyanate were highly skewed (Shapiro–Wilk, *p* > 0.05) with concentrations varying up to 1500-fold between participants. The median 24 h urinary concentrations for iodide, thiocyanate and nitrate were 111 μg/L (0.87 μmol/L, *n* = 800), 680 μg/L (11.7 μmol/L, *n* = 713), and 73.9 mg/L (587 μmol/L, *n* = 800), respectively. As creatinine normalization was not required with 24 h urine collection due to recording of total urine volume from each participant, the corresponding median daily amounts excreted for iodide, thiocyanate and nitrate were determined as 226 μg/day, 1.39 mg/day and 150 mg/day, respectively. Figure 1A,B summarizes the iodine status of PURE-24USE participants classified according to WHO guidelines [21] based on 24 h UIC (µg/L) and 24 h UIE (µg/day) metrics. Overall, the median UIC of 111 μg/L was within adequacy requirements (100–199 μg/L) with 11.9% of the population (<20%) having moderate (20–49 μg/L, 9.3%) or severe (<20 μg/L, 2.6%) iodine deficiency. Similar outcomes of adequate iodine nutrition in the population were evident when using more robust 24 h median UIE of 226 μg/day that was above the recommended dietary allowance (RDA of 150 μg/day) with a much lower fraction of the population categorized with moderate (2.1%) or severe (2.1%) iodine deficiency (<75 μg/day). In contrast, more participants had excessive iodine intake based on 24 h UIE (13.4%; ≥450 μg/day) as compared to 24 h UIC (7.3%; ≥300 μg/L) metrics. Figure 1C confirms that most participants (~ 91%) had a daily iodine intake (assuming 92% bioavailability) within an acceptable interval (95–1099 μg/day) with only 7.1% below EAR (<95 μg/day) and 1.8% greater than tolerable UL (≥1100 μg/day). 

### 3.3. Factors Contributing to Iodine Deficiency in Canada

Table 1 summarizes the major variables associated with iodine deficiency (<150 μg/day or <100 μg/L) when using a binary logistic regression model after adjustments for age, sex, BMI, total caloric intake, and diet quality (AHEI score). Overall, variables that were consistently protective against iodine deficiency (OR < 1.0, *p* < 0.05) using either UIE and UIC included use of iodine supplements, T4 prescription, site location, and dairy intake. Also, urinary sodium excretion was inversely associated with the risk of iodine deficiency based on UIE reflecting greater intake of iodized salt in foods. All other self-reports of salt intake (e.g., salty foods, table salt use at table and cooking, processed foods etc.) were not associated with iodine status. Age, as well as greater bread and cereal intake, were marginally protective against iodine deficiency based on UIC, whereas alcohol consumption increased risk for iodine deficiency. Interestingly, 24 h urine volume showed opposing trends likely reflecting a dilution effect when using UIC as a metric for iodine status resulting in an apparent risk for iodide deficiency. However, correction for differences in hydration based on UIE likely accurately reflects a true protective effect due to iodine uptake from greater daily drinking water/fluid consumption.

Other factors associated with iodine deficiency (OR > 1.0, *p* < 0.05) were site location, where residents from Vancouver and Quebec City had about a 2.5-fold greater relative risk as compared to Hamilton or Ottawa. Figure 1D shows box plots confirming that the median 24 h UIE for residents of Hamilton (264 μg/day, *n* = 198) and Ottawa (267 μg/day, *n* = 173) were higher than Vancouver (194 μg/day, *n* = 172) and Quebec City (191 μg/day, *n* = 194) based on ANCOVA after a Bonferroni correction and adjustments for age, sex, BMI, total caloric intake, AHEI score, education, smoking status and alcohol use (*F* = 8.80, *p* = 9.82 × 10^−6^, *n* = 737). In contrast, iodine supplement use (*F* = 42.3, *p* = 1.52 × 10^−10^, *n* = 681) without a T4 prescription had the greatest effect on iodine status when compared to T4 alone without iodine supplement use (*F* = 9.71, *p* = 1.91 × 10^−3^, *n* = 644) as illustrated in Figure 1E,F. Table S3 confirms that lower dairy and bread and cereal consumption were dietary patterns associated with iodine inadequacy when participants were categorized based on their iodine status as quintiles (Q1 vs. Q2–5) with a larger fraction from Vancouver and Quebec City, with fewer taking iodine supplements or T4 hormone therapy. Table S4 summarizes a Spearman rank correlation analysis of self-reported dietary intake of specific foods as a function of UIE (μg/day) after excluding participants using iodine supplements and/or T4. Overall, dairy intake had the strongest positive correlation (*r* = 0.24, *p* = 2.38 × 10^−9^, *n* = 611) with iodine status that was most evident for residents in Hamilton and Ottawa. In contrast, intake of bread and cereal, as well as processed food or a combination of red and processed meat were important sources related to iodine status for residents of Quebec City. Other potential iodine containing foods surveyed in this study, such as fish, eggs, and various vegetables, were not significant sources of dietary iodine.

### 3.4. Risk Assessment of Iodine Deficiency from Exposure to Environmental Iodine Uptake Inhibitors

**Figure 2:**
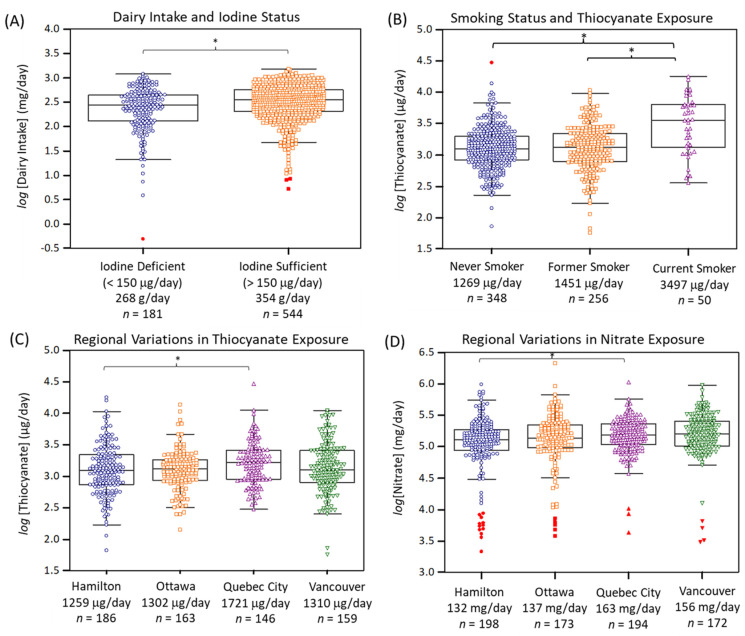
(**A**) Dairy intake was the most significant dietary exposure associated with iodine deficiency (*F* = 18.7, *p* = 1.75 × 10^−5^, *n* = 725), whereas (**B**) current smoking was a lifestyle factor associated with elevated urinary thiocyanate excretion as compared to former or never smokers (*F* = 19.5, *p* = 5.82 × 10^−9^, *n* = 654). Modest regional variations in exposure to environmental iodide uptake inhibitors, (**C**) thiocyanate and (**D**) nitrate were found. Overall, residents from Quebec City were exposed to both higher thiocyanate (*F* = 3.32, *p* = 0.0194, *n* = 654) and nitrate (*F* = 3.61, *p* = 0.0130, *n* = 737) relative to Hamilton when using ANCOVA after adjustment for covariates with a Bonferroni correction (* *p* < 0.05).

Figure 2A confirms that dairy consumption was an important dietary pattern associated with iodine deficiency in the PURE-24USE study (*F* = 18.7, *p* = 1.75 × 10^−5^, *n* = 725) as compared to 24 h sodium excretion (*F* = 16.9, *p* = 4.47 × 10^−5^, *n* = 737), and bread and cereal consumption (*F* = 1.44, *p* = 0.230, *n* = 728); the latter food source of iodine was only marginally significant when comparing iodine deficient vs. iodine sufficient participants in an unadjusted student’s t-test (*p* = 0.0551). Other environmental exposures may also modulate iodine deficiency risk despite adequate iodine nutrition. In this case, urinary thiocyanate and nitrate concentrations and their daily excretion amounts were also measured in this study. Overall, median urinary thiocyanate and nitrate concentrations were 6.1 and 670-fold higher than corresponding iodide levels. Figure 2B highlights that urinary thiocyanate was strongly dependent on smoking status (*F* = 19.5, *p* = 5.82 × 10^−9^, *n* = 654) with median 24 h thiocyanate excretion of about 1270 μg/day (*n* = 348), 1450 μg/day (*n* = 256), and 3500 μg/day (*n* = 50) corresponding to never smokers, former smokers, and current smokers, respectively. Otherwise, Table S5 highlights that dietary sources of thiocyanate (after excluding current smokers) were only weakly associated with intake of processed meat, cruciferous vegetables, and eggs (*r* ~ 0.10, *p* ~ 0.020, *n* = 620). 

Table S6 highlights that urinary nitrate had a moderate association to the intake of (total) vegetables (*r* = 0.17, *p* = 2.46 × 10^−6^, *n* = 759), as well as other specific vegetables (e.g., green leafy, cruciferous), and fruit. Indeed, there was a modest difference in 24 h urinary nitrate excretion when comparing low vs. high consumers of vegetables (*F* = 4.46, *p* = 0.0351, *n* = 728). However, there was no association of higher urinary nitrate excretion and lower blood pressure, nor was it related to hypertension prevalence [40]. There were regional variations in thiocyanate and nitrate exposures across Canada, although not as striking as for iodine status (Figure 1D). Figure 2C,D demonstrate that residents from Quebec City had greater exposure to both thiocyanate (*F* = 3.32, *p* = 0.0194, *n* = 654) and nitrate (*F* = 3.61, *p* = 0.0130, *n* = 737) relative to Hamilton, after covariate adjustments and Bonferroni correction. Also, residents from Vancouver had modestly elevated exposure to nitrate as compared to Hamilton in an unadjusted ANOVA model. 

## 4. Discussion

### 4.1. Iodine Nutritional Status of Canadian Adults 

Canada first introduced mandatory iodized salt for table or household use in 1949 as a prophylaxis to prevent iodine deficiency disorders due to the prevalence of iodine-deficient soils [41]. However, continuous monitoring of national programs is needed to optimize iodized salt content (~25 mg/kg) to ensure adequate nutrition in diverse populations with changing eating patterns [42]. A household iodine nutrition survey by the Canadian Health Measures Survey from 2007 to 2009 [29], as well as a recent study in children and young women in Canada [30], concluded adequate iodine nutrition in the population, including in high-risk demographic groups. The PURE-24USE study recently reported that daily sodium intake for Canadians was similar to other Western countries with about 47% of participants consuming <3 g/day [33] unlike other regions prone to excessive salt intake [11]. Thus, population data does not support sodium restriction as a public health policy for blood pressure reduction in Canada [43]. As a result, there is growing concern of the impact on iodine nutrition when promoting ‘heart-healthy’ salt-restricted diets, which may also include processed foods lacking iodized salt [44]. Additionally, vegans and vegetarians have greater risk for iodine deficiency with about half being below an EAR of 100 µg/day without iodine supplementation [45]. In fact, low iodine and selenium intake among vegans and vegetarian women represents a nutritional vulnerability [46]. To the best of our knowledge, our work is the first epidemiological study to examine iodine nutrition and exposure to iodine uptake inhibitors in Canadian adults (2012 to 2013) using a robust 24 h urine collection procedure for direct assessment of UIE [33]. In contrast, determination of iodine status from random spot urine samples is prone to significant day-to-day [47] and diurnal variations with peak concentrations excreted 4–5 h after main meals [48]. The CE assay used in this study offers a simple and low-cost microseparation platform compared to ion chromatography-tandem mass spectrometry [49] to differentiate urinary iodide from other iodine species (e.g., iodine, iodate etc.) while also allowing for the analysis of nitrate and thiocyanate [34,35] unlike inductive coupled plasma-mass spectrometry. 

We confirmed adequate iodine nutrition based on a median 24 h UIE of 226 μg/day and 24 h UIC of 111 μg/L with 4.2% and 11.9% categorized as moderately and severely deficient respectively based on WHO guidelines [21] in our cross-sectional study of community-dwelling participants living in four cities across Canada. However, dietary thresholds for estimating iodine status have assumed a mean 24 h urine volume of 1.5 L in adults. In this case, the impact of hydration status can overestimate the prevalence of iodide deficiency when relying on UIC as compared to UIE, which represents an age-old problem in urinalysis [50]. In our study, 7.1% of participants have sub-optimal iodine intake < EAR of 95 μg/day with few (1.8% > UL) prone to the deleterious effects from excessive iodine consumption of over-iodized salt, seaweeds, iodine supplements, medications, or a combination of these sources [13]. These two latter extreme conditions may be associated with adverse thyroid related health effects based on recommended Canadian dietary reference intakes [51]. However, thyroid function and other biomarkers of hyper-/hypothyroidism (e.g., thyroid-stimulating hormone) were not evaluated in the PURE-24USE study, which focused on the impact of dietary salt intake on blood pressure and hypertension [33].

Overall, use of iodine containing multivitamins was the single most important factor contributing to iodine adequacy in our study as compared to T4 use. For instance, the median UIE for individuals taking both iodine supplements and T4, iodine supplements alone (no T4), T4 hormone therapy alone (no iodine supplements) as compared to controls was 575 μg/day (*n* = 8), 360 μg/day (*n* = 95), 271 μg/day (*n* =53) and 206 μg/day (*n* = 644), respectively. The use of iodine supplements has been shown to be a strong predictor of iodine status as compared to other dietary sources [46], including in pregnant women [52]. Indeed, a large fraction of popular adult multivitamin products now contain iodine primarily as potassium iodide [53]. However, an analysis of prenatal multivitamins in the US market revealed that some products may contain more than three-times the recommended daily intake of iodine especially if derived from kelp [54]. T4 is a prescribed thyroid hormone often used for treatment of hypothyroidism due to a thyroid dysfunction impairing normal iodine uptake, such as Hashimoto’s thyroiditis. Yet, certain patients may be prescribed T4 for non-thyroid indications, such as treatment for fatigue or obesity [55]. Deiodination of T4 following ingestion and metabolism (average dosage ~ 125 μg) likely results in its preferential renal excretion as iodide [56] that increases UIE to a greater extent than typical food sources of iodine in the Canadian diet. However, simultaneous intake of T4 and iodine supplements is not recommended given concerns of excessive iodine intake with potential risks for hyperthyroidism. Otherwise, only two participants in this study were reported to be using another iodine containing prescribed medication, namely amiodarone. However, recent use of iodine containing contrast agents for diagnostic imaging was not included in the questionnaire.

### 4.2. Major Dietary Sources of Iodine Nutrition in Canada

Dairy intake was the most significant food source to differentiate iodine status (*p* = 1.75 × 10^−5^) in our cohort of Canadian adults after adjustment for covariates (Figure 2A) as compared to total salt intake (i.e., 24 h urinary sodium excretion), or bread and cereal, and processed food consumption. Milk and dairy products are important sources of iodine that contribute about 40% of total iodine nutrition in non-pregnant adults relative to about 11% for fish and seafood in the United Kingdom [57]. However, dairy may constitute a greater fraction of total dietary iodine in other western countries given its more frequent consumption than fish [52]. In fact, postmenopausal women with reduced milk intake are at greater risk for iodine deficiency as compared to daily milk consumers, despite regular iodized salt use [58]. A survey of Canadian dairy farms reported a variable iodine content in milk in different provinces, which was dependent on feeding and sanitation practices, such as spraying or dipping teats with iodophors before milking [59]. Thus, human iodine intake from milk and dairy products arises from cattle fodder and feed fortification, as well as indirectly via transdermal uptake and/or incidental contamination of iodine containing disinfectants during milking [60]. This scenario has been characterized as an accidental public health triumph for eliminating endemic goiter in Britain [61]. The median dairy intake for PURE participants was 333 g/day (ranging from 0 to 1520 g/day), which corresponds to a theoretical iodine intake of 102 μg/day or 68% of the recommended non-pregnant adult daily requirement assuming a mean iodine concentration of 304 μg/kg in Canadian milk [59]. Data from the USA reported that iodine content of retail milk products was variable with an average 85 μg/serving (240 mL) that did not depend on milk fat content while supplying about 57% of daily iodine intake [18]. Recent studies have also demonstrated that iodide is the predominant iodine species in cow milk that has high bioavailability, which is recommended for children and pregnant women given their higher iodine nutritional requirements [62]. However, there is growing risk for iodine deficiency due to increased consumption of unfortified milk alternative drinks derived from soya, oat, hemp, rice and various nuts (e.g., almond, coconut) that contain only 1.7% of cow milk’s iodine content [63]. Although most of these milk substitutes are fortified with calcium, few products are fortified with iodine.

Self-reported measures of salt use from questionnaires were not identified as significant source of dietary iodine in the PURE-24USE study with only processed food consumption being weakly correlated to UIE. On the other hand, daily urinary sodium excretion was found to differentiate iodine status after adjustment for covariates (*p* = 4.47 × 10^−5^). For instance, iodine deficient (<150 μg/day) as compared to sufficient (≥150 μg/day) participants with completed diet records (*n* = 737) had a median urinary sodium excretion of 2.74 g/day and 3.15 g/day, respectively. Overall, only 10% of participants had excessive sodium intake of ≥5 g/day with a median sodium intake of 3.08 g/day, which highlights that public health policies to restrict sodium intake in the population [44,45] may negatively impact universal iodized salt programs unless other iodine-rich sources of food are regularly consumed [13]. This policy conflict is reflected by recent changes to Canada’s Food Guide in 2020 that discourages animal protein consumption (i.e., milk and dairy) in favor of plant-based protein sources while also recommending meals to be prepared with little to no added salt [64]. Although fish consumption was not associated with iodine status in this study, bread and cereal intake was weakly protective against iodine deficiency albeit much less significant than dairy products, as well as iodine supplement or T4 use. This may reflect the declining use of iodate as a conditioner in bread and baking products in North America [12] in contrast to public health initiatives to fortify breads with iodized salt, such as in Australia [16] and Denmark [65].

### 4.3. Regional Variations in Iodine Deficiency across Canada Modulated by Exposure to Iodine Uptake Inhibitors

An unexpected result from our multi-center cross-sectional study was the variation in iodine status for PURE participants across the four Canadian study sites, as well as their differential exposure to environmental iodide uptake inhibitors. Similar regional variations in iodine status were reported in three cities in Turkey whose population is mildly iodine deficient [32]. Also, regional variations in iodine status have been reported across four cities in China with evidence of adequate iodine status [66]. Although there is iodine adequacy on a population level in Canada, residents from Quebec City and Vancouver (~191 μg/day or 92.1 μg/L, *n* = 400) were at a 2.5-fold greater relative risk for iodine deficiency as compared to Hamilton and Ottawa (~269 μg/day or 124 μg/L, *n* = 400) after covariate adjustments and Bonferroni correction. These regional variations in iodine status persisted after excluding for differential iodine supplement and T4 use. Yet, residents from Quebec City had the highest 24 h urinary sodium excretion (median of 3.70 g/day) and dairy intake (median of 379 g/day) relative to other sites, whereas Vancouver residents had average sodium excretion (median of 2.99 g/day) and a lower mean dairy consumption (median of 285 g/day). The variable iodine content of Canadian milk producers differs regionally [59] likely explains these discordant trends when relying on a standardized questionnaire for diary intake for participants from three different provinces, including Ontario, British Columbia, and Quebec. 

Indeed, there was a poor correlation between self-reported dairy intake and iodine status for residents from Quebec City and Vancouver, unlike Hamilton and Ottawa (both in Ontario) implying regional variations in milk iodine content. In contrast, the iodine status of residents from Quebec City had a weak correlation with bread and cereal intake, as well as processed food and processed meat consumption reflecting distinctive eating patterns despite no overall difference in diet quality (AHEI score). However, alcohol consumption weakly increased risk for iodine deficiency in the PURE-24USE study, which is not consistent with the association of moderate alcohol use and lower rates of goiter, single thyroid nodules and autoimmune hypothyroidism [67,68]. The analysis of iodine content in household drinking water from urban and rural sites in Canada may represent an important yet unexplained dietary iodine source not examined in this study. For instance, there is growing recognition of the importance of iodine-rich spring and ground water sources which may lead to excessive iodine intake in certain regions [69]. Indeed, there was over a ten-fold range in hydration status recorded among PURE participants with a median 24 h urine volume of 2.13 L (*n* = 800) ranging from 0.63 to 6.77 L that is corrected with UIE determination indicative of a protective effect against iodine deficiency due to greater daily fluid intake.

Although urinary perchlorate was not detected by the CE method, two abundant iodine uptake inhibitors were measured together with iodide, namely thiocyanate and nitrate. For instance, a median urinary perchlorate concentration of 3.2 μg/L was reported in pregnant women from Toronto, Canada that was about 100-fold lower than urinary thiocyanate [70]. Although thiocyanate is a weaker antagonist of the sodium-iodide symporter, the relative potency of perchlorate to inhibit iodide uptake is about 15- and 240-times that of thiocyanate and nitrate on a molar concentration basis [71]. Thus, the much higher concentrations of thiocyanate and nitrate render these ubiquitous thyroid antagonists essential when estimating their combined inhibitory effect based on a perchlorate equivalent concentration [72]. Although thiocyanate is biosynthesized in-vivo, exogenous sources are derived by smoke inhalation of hydrogen cyanide following combustion of nitrogen-containing tobacco alkaloids, and the digestion of certain goitrogenic foods, such as vegetables containing cyanogenic glycosides [73]. As expected, urinary thiocyanate excretion was strongly determined by smoking status [74] with smaller background dietary contributions from the intake of cruciferous vegetables and processed meat. Overall, there was a 2.6-fold (*p* = 5.82 × 10^−9^) greater thiocyanate excretion in current smokers as compared to former/never smokers. Women who are heavy smokers have been reported to be at greater risk for hypothyroxinemia from excessive thiocyanate exposures [75]. The median urinary thiocyanate excretion of 1395 μg/day (or 680 μg/L) was 67-fold greater than iodide with higher thiocyanate exposures measured for residents in Quebec City as compared to Hamilton, Ottawa, and Vancouver after covariate adjustment. 

Similarly, residents of Quebec City also had modestly higher exposure to nitrate relative to Hamilton. Overall, there was a 664-fold higher urinary nitrate concentration than iodide with a median 24 h excretion of 150 mg/day (or 7.41 mg/L, *n* = 800). Unlike thiocyanate, urinary nitrate was unrelated to tobacco smoking, and it had a stronger correlation to dietary intake of vegetables, notably green leafy vegetables. However, residents from Quebec City also had nitrate exposures from processed meat and fruit intake unlike other study sites (Table S5). Similar to iodide, drinking water is likely an unaccounted source of nitrate exposures [76] in the PURE-24USE study, which may be acute in ground water contaminated by agricultural fertilizer run-off. Although greater nitrate exposure may have putative health benefits to reduce hypertension and cardiovascular disease risk reflecting higher nitric oxide levels [41], there was no significant correlation to blood pressure (systolic or diastolic) or hypertension incidence in this study. Nonetheless, nitrate and thiocyanate are anticipated to have a 96-fold and 14-fold greater inhibitory effect on iodide uptake as compared to reported urinary perchlorate levels [71] given their much higher exposure levels consistent with previous risk assessment calculations [77]. Consequently, residents from Vancouver and especially Quebec City may be at greater relative risk for iodine deficiency due to their suboptimal iodine nutritional status and greater combined exposures to thiocyanate and/or nitrate.

### 4.4. Study Strengths, Limitations and Future Perspectives

Major strengths of this study include the use of a robust 24 h urine collection procedure together with a coordinated 24 h dietary questionnaire timed during specimen drop-off at four different clinical sites across Canada. A validated CE method was also used for quantitative iodide, thiocyanate and nitrate determination in urine directly after a simple dilution step. Moreover, complementary statistical methods were adjusted for potential confounders with key outcomes remaining robust relative to unadjusted models. Limitations include that this cross-sectional study was not representative of Canadians given selection criteria for participant selection was focused on sodium and potassium intake primarily in older persons, which excluded children and pregnant women [33]. Even though total urine volume was used to correct for variable hydration status when reporting iodine status as UIE, the recording of daily volume of water or beverages ingested, source(s) of water (e.g., tap, bottle), as well as iodide content analyses of local drinking water and commercial milk products in different sites were also study limitations. A more detailed food frequency questionnaire that included specific iodine-rich foods, such as seaweed/marine algae, and ocean fish/seafood, as well as adherence to increasingly popular diets (e.g., vegetarian, vegan, lactose-intolerant, ketogenic, low-salt, paleolithic) is recommended in future studies given their likely impact on iodine status [78]. Although nitrate exposure was not related to hypertension in this study, iodine deficiency with low urinary iodide levels is associated with hyperlipidemia and greater cardiovascular disease risk highlighting the broader public health benefits of optimal iodine prophylaxis [79,80]. Future studies involving iodine nutrition in older persons would benefit from biochemical measures of thyroid function, blood lipid panels, and inflammatory biomarkers during clinical visits. Direct analysis of circulating levels of iodide in serum may also provide deeper insights into iodine status and thyroid function than urine biomonitoring [66]. Lastly, national food guidelines recommending salt-restricted foods and plant-based protein substitutes warrant further scrutiny to the potential negative impacts on iodine deficiency in susceptible populations without iodine supplementation or mandatory iodine fortification of staple foods (e.g., bread) and commercial products (e.g., milk substitutes).

## 5. Conclusions

In summary, the iodine status of Canadian adults (*n* = 800) surveyed from 2012 to 2013 was determined to be adequate on a population level with a low prevalence of moderate to mild deficiency or excessive iodine intake. Overall, 24 h UIE provided a more robust indicator of iodine status than UIC that allowed for direct assessment of dietary reference intervals without creatinine adjustments. Iodine supplement use, T4 prescription, dairy intake, 24 h sodium excretion, and 24 h urine volume were key protective factors against iodine deficiency in this study. On the other hand, residents from Quebec City and Vancouver were at greater risk for iodine deficiency than Hamilton and Ottawa. These regional differences in iodine status may be further exacerbated by greater exposure to thiocyanate and nitrate as ubiquitous iodine uptake inhibitors. Continued iodine surveillance is warranted given greater consumption of processed foods, increased popularity of salt-restricted and other specialized diets, as well as emerging environmental exposures that may increase iodine deficiency risk in Canada. This work highlights that national level iodine adequacy may obscure regional differences in iodine status in local populations. Greater public awareness of the importance of optimal iodine nutrition in a healthy diet is strongly recommended along with public health guidelines that better align the optimal dietary intakes of sodium and iodine in the population. 

## Figures and Tables

**Table 1:** Protective and risk factors for iodine deficiency (<150 µg/day or <100 µg/L) among PURE-24USE participants (*n* = 800) using a binary linear logistic regression. Significant variables (*p* < 0.05) are bolded after adjustments for age, sex, BMI, total caloric intake and AHEI score.

Variable	24 h UIE (µg/day)		24 h UIC (µg/L)	
	OR (95% CI)	*p*-Value	OR (95% CI)	*p*-Value
Age	0.99 (0.99–1.01)	0.257	**0.98 (0.97–1.00)**	**0.0516**
Male sex	0.80 (0.56–1.14)	0.215	0.76 (0.56–1.03)	0.0754
BMI (>27 kg/m^2^)	**0.66 (0.47–0.92)**	**0.0153**	0.99 (0.96–1.02)	0.515
24 h Urine volume (L)	**0.69 (0.56–0.85)**	**4.07 × 10^−4^**	**2.31 (1.91–2.81)**	**5.87 × 10^−17^**
Current smoker	0.85 (0.43–1.67)	0.635	1.13 (0.64–1.99)	0.671
Current alcohol consumer	1.59 (0.98–2.47)	0.059	1.48 (1.00–2.20)	0.0505
Study site:				
Hamilton	1.00 (ref.)	--	1.00 (ref.)	--
Vancouver	**2.54 (1.52–4.23)**	**3.57 × 10^−4^**	**1.83 (1.20–2.81)**	**5.31 × 10^−3^**
Quebec City	**2.58 (1.57–4.22)**	**1.74 × 10^−4^**	**1.89 (1.25–2.84)**	**2.41 × 10^−3^**
Ottawa	1.19 (0.69–2.05)	0.531	1.28 (0.85–1.93)	0.839
Rural location	0.99 (0.69–1.60)	0.953	1.28 (0.85–1.93)	0.246
Iodine supplementation	**0.18 (0.08–0.41)**	**6.30 × 10^−5^**	**0.31 (0.19–0.52)**	**8.77 × 10^−6^**
T4 prescription	**0.33 (0.14–0.78)**	**1.20 × 10^−2^**	**0.43 (0.23–0.79)**	**6.76 × 10^−3^**
Dairy intake (g/day)	**0.999 (0.998–0.999)**	**3.94 × 10^−4^**	**0.999 (0.998–1.00)**	**4.41 × 10^−4^**
Starch intake (g/day)	0.999 (0.997–1.00)	0.105	**0.999 (0.997–1.00)**	**0.0470**
Sodium excretion (g/day)	**0.71 (0.61–0.84)**	**3.03 × 10^−5^**	0.93 (0.83–1.05)	0.265
Salty food intake (g/day)	1.00 (1.00–1.01)	0.108	1.00 (0.97–1.00)	0.856

CI = confidence interval, OR = odds ratios, ref. = reference. Hosmer–Lemeshow goodness-of-fit logistic regression, UIE, urinary iodine excretion; UIC, urinary iodine concentration. Statistically significant variables (*p* < 0.05) are bolded.

## Data Availability

The data presented in this study are available on request from the corresponding author. The data are not publicly available due to participant privacy.

## Supplementary Materials

The following are available online at https://www.mdpi.com/article/10.3390/nu14132570/s1, Table S1: Summary of characteristics of PURE-24USE cohort; Table S2: Summary of figures of merit of CE assay for determination of urinary iodide, nitrate and thiocyanate; Table S3: Characteristics of participants categorized by quintiles based on 24 h UIE; Table S4: Spearman rank correlation analysis of dietary variables associated with daily iodine excretion; Table S5: Spearman rank correlation analysis of dietary variables associated with daily thiocyanate excretion; Table S6: Spearman rank correlation analysis of dietary variables associated with daily nitrate excretion; Figure S1: Representative electropherogram overlay and control charts for reliable quantification of iodide, nitrate and thiocyanate in 24 h urine samples.
