# Supplementary material for: The Prevalence and Risk Factors Associated with Iodine Deficiency in Canadian Adults
Source: Nutrients. 2022 Jun 21;14(13):2570. doi: 10.3390/nu14132570 (PMC9268597; doi:10.3390/nu14132570)
Supplement: Supplementary file 1 [file nutrients-14-02570-s001.zip › nutrients-1776550-supplementary.pdf]

# SUPPLEMENTAL INFORMATION

# The Prevalence and Risk Factors Associated with Iodine Deficiency in Canadian Adults

Stellena Mathiaparanam,[1] Adriana Nori de Macedo,[1,2] Andrew Mente,[3] Paul Poirier,[4] Scott A. Lear,[5] Andreas Wielgosz,[6] Koon Teo,[3] Salim Yusuf,[3] Philip Britz-McKibbin[1]*

[1] *Department of Chemistry and Chemical Biology, McMaster University, Hamilton, ON, Canada*
[2] *Departamento de Química, Universidade Federal de Minas Gerais, Belo Horizonte, Minas Gerais, Brazil*
[3] *Population Health Research Institute, McMaster University and Hamilton Health Sciences, Hamilton, Ontario, Canada*
[4] *Faculté de Pharmacie, Institut Universitaire de Cardiologie et de Pneumologie de Québec, Université Laval, Québec City, Québec, Canada*
[5] *Faculty of Health Sciences, Simon Fraser University, Burnaby and Division of Cardiology, Providence Health Care, Vancouver, British Columbia, Canada*
[6] *University of Ottawa Heart Institute, Ottawa, Ontario, Canada*

* Corresponding author: Philip Britz-McKibbin, britz@mcmaster.ca

**Table S1-Table S6, Figure S1**

**Table S1.** Characteristics of PURE-24USE participants categorized by study regions, including Hamilton, Vancouver, Quebec City and Ottawa.

| **Predictor Variable** | **Study Site** | | | | |
|---|---|---|---|---|---|
| | **Hamilton (n=217)** | **Vancouver (n=200)** | **Quebec City (n=200)** | **Ottawa (n=183)** | **Total (n=800)** |
| 24 h iodine concentration (μg/L) – median ± IQR | 122 ± 115 | 91 ± 84 | 95 ± 87 | 128 ± 106 | 111 ± 98 |
| 24 h daily iodine excretion (μg/day) – median ± IQR | 272 ± 190 | 194 ±170 | 191 ±142 | 267 ± 242 | 226 ± 191 |
| 24 h thiocyanate concentration (μg/L) – median ± IQR[a] | 658 ± 779 | 649 ± 656 | 846 ± 841 | 668 ± 904 | 680 ± 811 |
| 24 h daily thiocyanate excretion (mg/day) – median ± IQR[a] | 1.3 ± 1.5 | 1.3 ± 1.0 | 1.7 ± 1.8 | 1.4 ± 1.9 | 1.4 ± 1.5 |
| 24 h nitrate concentration (mg/L) – median ± IQR | 67 ± 57 | 79 ± 72 | 82 ± 58 | 67 ± 71 | 74 ± 64 |
| 24 h daily nitrate excretion (mg/day) – median ± IQR | 133 ± 104 | 167 ± 154 | 164 ± 123 | 137 ± 132 | 150 ± 126 |
| 24 h sodium concentration (g/L) – median ± IQR | 1.5 ± 1.1 | 1.3 ± 1.1 | 1.8 ± 1.2 | 1.3 ± 1.0 | 1.4 ± 1.1 |
| 24 h daily sodium excretion (g/day) – median ± IQR | 3.0 ± 1.6 | 3.0 ± 1.7 | 3.7 ± 2.0 | 2.7 ± 1.4 | 3.1 ± 1.7 |
| Age – years, – median ± IQR | 62 ± 13 | 59 ± 12 | 59 ± 14 | 64 ± 13 | 61 ± 12 |
| BMI (kg/m$^2$) – median ± IQR[b] | 28 ± 7.0 | 26 ± 6.9 | 27 ± 6.4 | 27 ± 6.2 | 27 ± 6.7 |
| Sex – female:male | 113:104 | 103:97 | 96:104 | 100:83 | 412:388 |
| 24 h urine volume (L) | 2.1 ± 1.2 | 2.2 ± 1.1 | 2.1 ± 1.2 | 2.3 ± 1.4 | 2.1 ± 1.2 |
| Iodine supplement – no./total no. (%) | 41/217 (19) | 31/200 (16) | 3/200 (2) | 28/183 (15) | 103/800 (13) |
| Thyroxine (T4) intake – no./total no. (%) | 11/217 (5) | 11/200 (6) | 21/200 (11) | 18/183 (10) | 61/800 (8) |
| Hypertension/high blood pressure diagnosis – no./total no. (%) | 52/217 (24) | 35/200 (18) | 36/200 (18) | 44/183 (24) | 167/800 (21) |
| Dairy intake (g/day) – median ± IQR[c] | 345 ± 359 | 285 ± 330 | 379 ± 397 | 320 ± 321 | 329 ± 359 |
| Starch intake (g/day) – median ± IQR[c] | 299 ± 210 | 290 ± 193 | 299 ± 192 | 314 ± 237 | 299 ± 208 |
| Sodium intake from ffq (mg) – median ± IQR[d] | 2.8 ± 1.4 | 2.5 ± 1.3 | 3.0 ± 1.4 | 2.7 ± 1.2 | 2.7 ± 1.3 |
| Current alcohol consumer – no./total no. (%) | 176/217 (81) | 152/200 (76) | 170/200 (85) | 155/183 (85) | 653/800 (82) |
| Current smoker – no./total no. (%) | 19/216 (9) | 12/200 (6) | 16/200 (8) | 12/179 (7) | 59/800 (7) |
| AHEI Score[e] | 38 ± 11 | 42 ± 14 | 37 ± 12 | 41 ± 14 | 39 ± 13 |
| Location – urban:rural | 211:6 | 186:14 | 106:94 | 182:1 | 685:115 |

[a] *Hamilton: n=204, Vancouver: n=184, Quebec City: n=152, Ottawa: n=173, and total: n=713.*
[b] *Hamilton: n=204, Vancouver: n=198, Quebec City: n=195, Ottawa: n=179, and total: n=776.*
[c] *Hamilton: n=207, Vancouver: n=173, Quebec City: n=197, Ottawa: n=182, and total: n=759.*
[d] *Hamilton: n=213, Vancouver: n=174, Quebec City: n=199, Ottawa: n=181, and total: n=767.*
[e] *Hamilton: n=212, Vancouver: n=174, Quebec City: n=199, Ottawa: n=181, and total: n=766.*

**Table S2.** Summary of figures of merit of CE assay for the determination of urinary iodide, nitrate and thiocyanate from PURE-24USE study participants (n=800).

| Figures of Merit | Iodide[a] | Nitrate[b] | Thiocyanate[c] |
|---|---|---|---|
| LOD (S/N = 3) | 0.020 µmol/L | 0.64 µmol/L | 0.12 µmol/L |
| LOQ (S/N = 10) | 0.070 µmol/L | 2.12 µmol/L | 0.40 µmol/L |
| Linearity ($R^2$) | 0.996 | 0.999 | 0.997 |
| Sensitivity (µmol/L)$^{-1}$ | 0.045 | 0.006 | 0.014 |
| Reproducibility (CV)[d] | 5.7% | 7.7% | 5.3% |
| Missing data/Non-detects[e] | 2.0% | 0% | 11% |

[a] *Calibration curve was normalized to NDS (20 µmol/L) over a 17-fold (6 calibrants) linear dynamic range, respectively. Calibrant concentrations were 0.15, 0.20, 0.50, 1.00, 1.80, 2.60 µmol/L.*

[b] *Calibration curve was normalized to NDS (20 µmol/L) over a 400-fold (6 calibrants) linear dynamic range, respectively. Calibrant concentrations were 0, 50, 100, 200, 300, 400 µmol/L.*

[c] *Calibration curve was normalized to NDS (20 µmol/L) over a 24-fold (6 calibrants) linear dynamic range, respectively. Calibrant concentrations were 0.5, 1.0, 2.0, 4.0, 8.0, 12.0 µmol/L.*

[d] *Reproducibility was assessed based on repeated analysis of a pooled urine samples from PURE as QC every batch of 10 runs.*

[e] *Missing data due to concentrations below method detection limit or matrix spectral interferences from PURE cohort (n=800).*

**Table S3.** Characteristics of PURE-24USE participants categorized by quintiles using 24 h UIE (μg/day).

| **Predictor Variable** | **Quintiles** | |
|---|---|---|
| | **Q1 (<133.5 μg/day; n=160)** | **Q2-5 (≥133.5 μg/day; n=640)** |
| 24 h daily iodine excretion (μg/day) – median ± IQR | 97 ± 40 | 263 ± 184 |
| 24 h daily thiocyanate excretion (mg/day) – median ± IQR[a] | 1.1 ± 1.0 | 1.5 ± 1.6 |
| 24 h daily nitrate excretion (mg/day) – median ± IQR | 148 ± 139 | 151 ± 122 |
| 24 h daily sodium excretion (g/day) – median ± IQR | 2.7 ± 1.5 | 3.2 ± 1.7 |
| Age – years, – median ± IQR | 60 ± 13 | 61 ± 13 |
| BMI (kg/m$^2$) – median ± IQR[b] | 26 ± 7 | 27 ± 6 |
| Sex – female:male | 88:72 | 324:316 |
| 24 h urine volume (L) | 1.9 ± 1.3 | 2.2 ± 1.1 |
| **Iodine supplement – no./total no. (%)** | **6/160 (4)** | **97/640 (15)** |
| **Thyroxine (T4) intake – no./total no. (%)** | **4/160 (3)** | **57/640 (9)** |
| **Dairy intake (g/day) – median ± IQR[c]** | **246 ± 303** | **352 ± 368** |
| **Starch intake (g/day) – median ± IQR[c]** | **286 ± 164** | **311 ± 217** |
| *Study City:* | | |
| **Hamilton – no./total no. (%)** | **29/160 (18)** | **188/640 (29)** |
| **Vancouver – no./total no. (%)** | **59/160 (37)** | **141/640 (22)** |
| **Quebec City – no./total no. (%)** | **46/160 (29)** | **154/640 (24)** |
| **Ottawa – no./total no. (%)** | **26/160 (16)** | **157/640 (25)** |
| Sodium intake from FFQ (g)[d] – median ± IQR[d] | 2.6 ± 1.2 | 2.8 ± 1.3 |
| Current alcohol consumer – no./total no. (%) | 136/160 (85) | 517/640 (81) |
| Current smoker – no./total no. (%) | 11/159 (7) | 48/636 (8) |
| AHEI Score[e] | 40 ± 14 | 39 ± 13 |
| Location – urban:rural | 138:22 | 547:93 |

[a] *Q1: n=135 and Q2-5: n=578.*
[b] *Q1: n=154 and Q2-5: n=622.*
[c] *Q1: n=150 and Q2-5: n=609.*
[d] *Q1: n=149 and Q2-5: n=618.*
[e] *Q1: n=149 and Q2-5: n=617.*

**Table S4.** Spearman rank correlation analysis of dietary variables associated with daily iodine excretion (µg/day) of PURE-24USE participants from the four different sites in Canada after excluding for iodine supplement and/or T4 use.

| **Dietary Intake** | **PURE Study Site** | | | | | | | | **Total (224 µg/day; *n*=611)** | |
|---|---|---|---|---|---|---|---|---|---|---|
| | **Hamilton (257 µg/day; *n*=162)** | | **Vancouver (189 µg/day; *n*=137)** | | **Quebec City (191 µg/day; *n*=173)** | | **Ottawa (260 µg/day; *n*=139)** | | | |
| | ***r*** | ***p*-value** | ***r*** | ***p*-value** | ***r*** | ***p*-value** | ***r*** | ***p*-value** | ***r*** | ***p*-value** |
| Dairy (g/day) | **0.39**** | **$2.15\times10^{-7}$** | 0.15 | $7.30\times10^{-2}$ | 0.09 | 0.266 | **0.34**** | **$4.33\times10^{-5}$** | **0.24**** | **$2.38\times10^{-9}$** |
| Bread and cereal (g/day) | **0.18*** | **$2.02\times10^{-2}$** | 0.002 | 0.984 | **0.15*** | **$5.31\times10^{-2}$** | 0.05 | 0.586 | **0.10*** | **$1.63\times10^{-2}$** |
| Processed food (g/day) | 0.05 | 0.565 | 0.01 | 0.953 | **0.22**** | **$4.22\times10^{-3}$** | 0.04 | 0.669 | **0.11**** | **$7.93\times10^{-3}$** |
| Red and proc. meat (g/day) | 0.10 | 0.208 | 0.07 | 0.447 | **0.16*** | **$3.97\times10^{-2}$** | 0.01 | 0.902 | 0.06 | 0.151 |
| Red meat only (g/day) | 0.13 | $9.66\times10^{-2}$ | 0.08 | 0.338 | 0.14 | $6.05\times10^{-2}$ | -0.01 | 0.946 | 0.06 | 0.171 |
| Processed meat (g/day) | -0.07 | 0.378 | -0.04 | 0.613 | 0.12 | 0.118 | 0.06 | 0.508 | 0.02 | 0.679 |
| White meat only (g/day) | 0.03 | 0.679 | 0.03 | 0.701 | 0.04 | 0.569 | -0.03 | 0.746 | 0.02 | 0.607 |
| Vegetable (g/day) | **0.16*** | **$4.88\times10^{-2}$** | -0.07 | 0.440 | -0.01 | 0.910 | 0.04 | 0.686 | 0.01 | 0.802 |
| Green leafy veg. (g/day) | 0.12 | 0.125 | -0.16 | $5.92\times10^{-2}$ | -0.08 | 0.318 | 0.05 | 0.602 | -0.004 | 0.923 |
| Cruciferous veg. (g/day) | 0.11 | 0.156 | 0.02 | 0.779 | 0.08 | 0.316 | 0.07 | 0.437 | 0.03 | 0.467 |
| Dark leafy veg. (g/day) | 0.07 | 0.376 | -0.07 | 0.415 | 0.02 | 0.783 | -0.02 | 0.853 | -0.01 | 0.800 |
| Egg (g/day) | 0.05 | 0.568 | -0.02 | 0.842 | 0.12 | 0.129 | -0.08 | 0.345 | 0.05 | 0.239 |
| Fruit (g/day) | 0.04 | 0.605 | -0.03 | 0.729 | 0.004 | 0.961 | -0.09 | 0.270 | -0.02 | 0.676 |
| Fish (g/day) | -0.03 | 0.686 | -0.06 | 0.454 | 0.02 | 0.812 | 0.02 | 0.796 | -0.02 | 0.709 |
| Salty food (g/day)[a] | -0.01 | 0.914 | -0.14 | 0.107 | 0.10 | 0.183 | -0.02 | 0.844 | 0.004 | 0.925 |

*Participants taking iodine containing supplements and thyroxine (T4) were excluded from the analysis, where r denotes the Spearman rank correlation coefficient.*

***Correlation is significant at the 0.01 level (2-tailed).*

** Correlation is significant at the 0.05 level (2-tailed).*

**Table S5.** Spearman rank correlation analysis of dietary variables associated with daily thiocyanate excretion (µg/day) of participants across four different sites in the PURE-24USE study after excluding for current smokers, including Hamilton, Vancouver, Quebec City and Ottawa.

| **Dietary Variable** | **Study Site** | | | | | | | | **Total (median=1354 µg/day; *n*=620)** | |
|---|---|---|---|---|---|---|---|---|---|---|
| | **Hamilton (median=1251 µg/day; *n*=176)** | | **Vancouver (median=1272 µg/day; *n*=152)** | | **Quebec City (median=1718 µg/day; *n*=136)** | | **Ottawa (median=1281 µg/day; *n*=156)** | | | |
| | ***r*** | ***p*-value** | ***r*** | ***p*-value** | ***r*** | ***p*-value** | ***r*** | ***p*-value** | ***r*** | ***p*-value** |
| Processed meat intake (g/day) | 0.04 | 0.645 | 0.04 | 0.642 | 0.16 | $6.53\times10^{-2}$ | 0.12 | 0.136 | **0.10*** | **$1.85\times10^{-2}$** |
| Red and processed meat intake (g/day) | 0.04 | 0.613 | 0.09 | 0.280 | -0.01 | 0.889 | 0.13 | 0.108 | **0.09*** | **$2.03\times10^{-2}$** |
| Cruciferous vegetable intake (g/day) | 0.11 | 0.147 | 0.13 | 0.103 | 0.01 | 0.917 | 0.05 | 0.541 | **0.09*** | **$2.08\times10^{-2}$** |
| Egg intake (g/day) | 0.13 | $8.18\times10^{-2}$ | 0.08 | 0.352 | **0.18*** | **$3.86\times10^{-2}$** | 0.05 | 0.512 | **0.09*** | **$3.30\times10^{-2}$** |
| Starch (g/day) | 0.03 | 0.743 | 0.05 | 0.582 | -0.09 | 0.305 | **0.16*** | **$4.47\times10^{-2}$** | 0.050 | 0.232 |

*Participants who self-reported as currently smoking were excluded from the analysis, where r denotes the Spearman rank correlation coefficient.*

***Correlation is significant at the 0.01 level (2-tailed).*

** Correlation is significant at the 0.05 level (2-tailed).*

*Significant p-values at the 0.01 and 0.05 levels are bolded.*

**Table S6.** Spearman rank correlation analysis of dietary variables associated with daily nitrate excretion (mg/day) of participants across from four different sites in the PURE-24USE study, including Hamilton, Vancouver, Quebec City and Ottawa.

| Dietary Variable | Study Site | | | | | | | | Total (median=147 mg/day; *n*=759) | |
|---|---|---|---|---|---|---|---|---|---|---|
| | Hamilton (median=133 mg/day; *n*=207) | | Vancouver (median=157 mg/day; *n*=173) | | Quebec City (median=162 mg/day; *n*=197) | | Ottawa (median=137 mg/day; *n*=182) | | | |
| | ***r*** | ***p*-value** | ***r*** | ***p*-value** | ***r*** | ***p*-value** | ***r*** | ***p*-value** | ***r*** | ***p*-value** |
| Vegetable intake (g/day) | **0.27**** | **$1.14\times10^{-4}$** | 0.09 | 0.217 | 0.14 | $5.90\times10^{-2}$ | 0.10 | 0.163 | **0.17**** | **$2.46\times10^{-6}$** |
| Green leafy vegetable intake (g/day) | **0.25**** | **$3.42\times10^{-4}$** | 0.10 | 0.173 | **0.14*** | **$4.86\times10^{-2}$** | 0.11 | 0.126 | **0.15**** | **$2.90\times10^{-5}$** |
| Other vegetable intake (g/day) | **0.16*** | **$2.25\times10^{-2}$** | 0.07 | 0.359 | 0.12 | $9.69\times10^{-2}$ | 0.03 | 0.675 | **0.11**** | **$2.77\times10^{-3}$** |
| Cruciferous vegetable intake (g/day) | **0.19**** | **$6.68\times10^{-3}$** | -0.06 | 0.399 | 0.01 | 0.866 | 0.06 | 0.393 | **0.07*** | **$4.99\times10^{-2}$** |
| Fruit intake (g/day) | 0.13 | $6.49\times10^{-2}$ | -0.04 | 0.646 | **0.19**** | **$7.50\times10^{-3}$** | 0.01 | 0.891 | **0.07*** | **$4.20\times10^{-2}$** |
| Processed meat intake (g/day) | **-0.18**** | **$9.37\times10^{-3}$** | -0.09 | 0.228 | **0.21**** | **$3.70\times10^{-3}$** | -0.003 | 0.963 | -0.02 | 0.600 |
| Processed food intake (g/day) | **-0.17*** | **$1.76\times10^{-2}$** | -0.02 | 0.812 | 0.04 | 0.540 | -0.07 | 0.355 | **-0.08*** | **$3.97\times10^{-2}$** |
| Dark yellow vegetable intake (g/day) | **0.16*** | **$1.83\times10^{-2}$** | 0.03 | 0.734 | 0.02 | 0.811 | -0.06 | 0.417 | 0.06 | 0.105 |
| Starch intake (g/day) | 0.08 | 0.266 | -0.10 | 0.191 | 0.04 | 0.603 | **0.19**** | **$8.94\times10^{-3}$** | 0.05 | 0.148 |

*r denotes the Spearman rank correlation coefficient.*

***Correlation is significant at the 0.01 level (2-tailed).*

** Correlation is significant at the 0.05 level (2-tailed).*

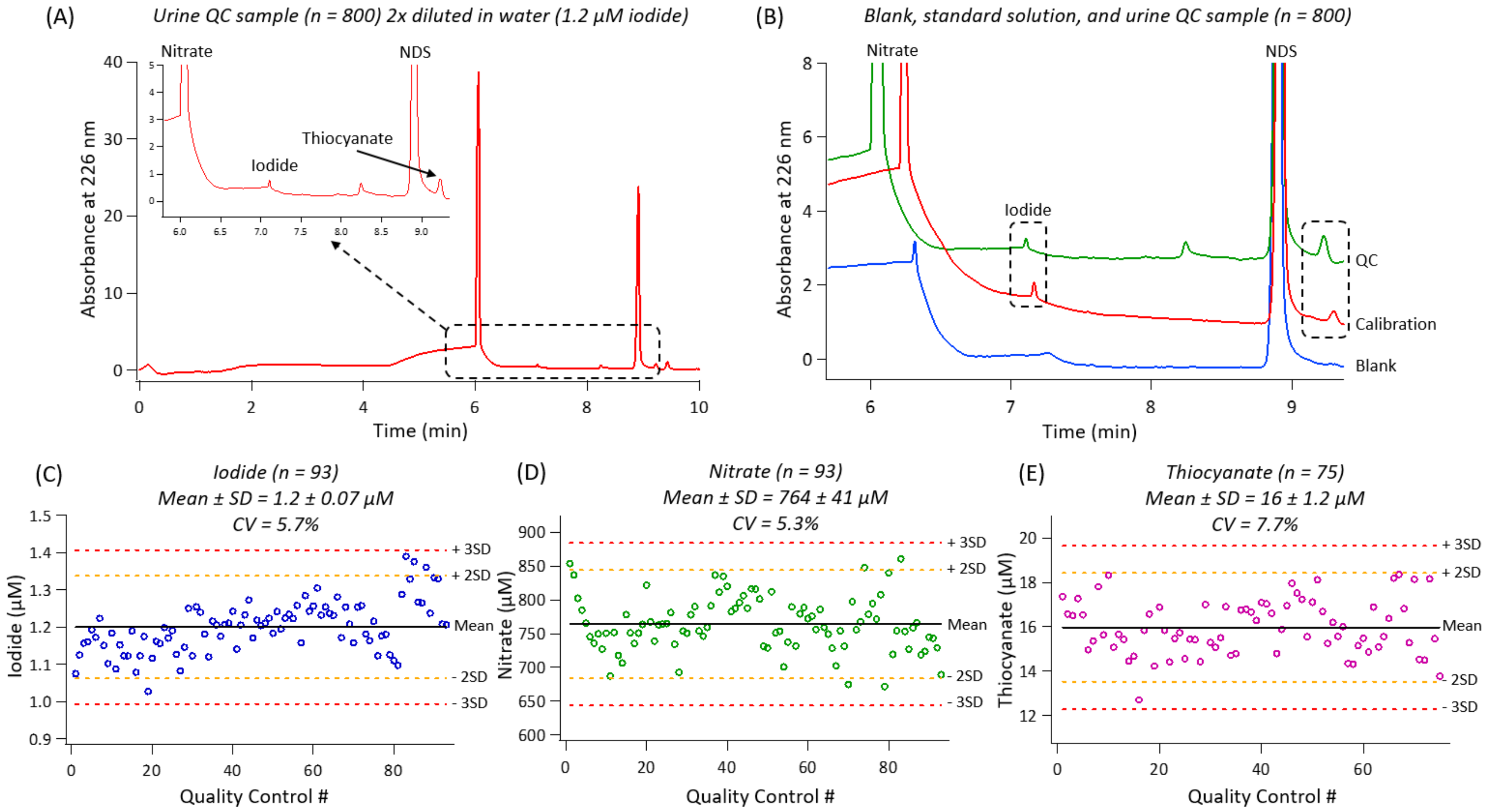


**Figure S1.** Representative electropherogram overlay of (A) a pooled quality control (*n*=800) and a comparison between a (B) quality control sample, calibrant solution, and blank solution. Also, control charts highlight reliable quantification of (C) iodide, (D) nitrate and (E) thiocyanate in 24 h urine samples with acceptable reproducibility (CV < 8%).
